# *N*-Butylphthalide Improves Cognitive Function in Rats after Carbon Monoxide Poisoning

**DOI:** 10.3389/fphar.2017.00064

**Published:** 2017-02-09

**Authors:** Ming-Jun Bi, Xian-Ni Sun, Yong Zou, Xiao-Yu Ding, Bin Liu, Yue-Heng Zhang, Da-Dong Guo, Qin Li

**Affiliations:** ^1^Department of Integration of Chinese and Western Medicine, The Affiliated Yantai Yuhuangding Hospital of Qingdao UniversityYantai, China; ^2^Emergency Centre, The Affiliated Yantai Yuhuangding Hospital of Qingdao UniversityYantai, China; ^3^Department of Integration of Chinese and Western Clinical Medicine, Qingdao University Medical CollegeQingdao, China; ^4^The Second Clinical Medical College, Shandong University of Traditional Chinese MedicineJinan, China; ^5^Department of Clinical Medicine, Binzhou Medical UniversityYantai, China; ^6^Eye Institute, Shandong University of Traditional Chinese MedicineJinan, China

**Keywords:** Ca^2+^/calmodulin dependent protein kinase II, Calpain 1, CO poisoning, cognitive function, *N*-butylphthalide, rat

## Abstract

Cognitive impairment is the most common neurologic sequelae after carbon monoxide (CO) poisoning, and the previous investigations have demonstrated that *N*-Butylphthalide (NBP) could exert a broad spectrum of neuroprotective properties. The current study aimed to investigate the effect of NBP on cognitive dysfunction in rats after acute severe CO poisoning. Rats were randomly divided into a normal control group, a CO poisoning group and a CO+NBP group. The animal model of CO poisoning was established by exposure to CO in a chamber, and then all rats received hyperbaric oxygen therapy once daily, while rats in CO+NBP group were administered orally NBP (6 mg/ 100g) by gavage twice a day additionally. The results indicated that CO poisoning could induce cognitive impairment. The ultrastructure of hippocampus was seriously damaged under transmission electron microscopy, and the expressions of calpain 1 and CaMK II proteins were significantly elevated after CO exposure according to the analysis of immunofluorescence staining and western blot. NBP treatment could evidently improve cognitive function, and maintain ultrastructure integrity of hippocampus. The expression levels of both calpain 1 and CaMK II proteins in CO+NBP group were considerably lower than that of CO poisoning group (*P* < 0.05). Taken together, this study highlights the molecular mechanism of cognitive dysfunction in rats after CO exposure via the upregulation of both calpain 1 and CaMK II proteins. The administration of NBP could balance the expressions of calpain 1 and CaMK II proteins and improve cognitive function through maintaining ultrastructural integrity of hippocampus, and thus may play a neuroprotective role in brain tissue in rats with CO poisoning.

## Introduction

From a public health perspective, unintentional carbon monoxide (CO) poisoning is a leading factor of accidental poisoning in the United States, and may be the cause of more than 50% fatal poisonings in many industrial countries (Omaye, 2002; Geraldo et al., 2014). The clinical signs and symptoms associated with CO toxicity effects depend on the concentration and duration of exposure, ranging from slight headache, nausea, vomiting, shortness of breath, malaise and palpitation, to confusion, unconsciousness, coma and even death (Wright, 2002). There are permanent neurologic problems in 46% of survivor (Choi, 2002; Yogaratnam et al., 2011), and the delayed neurological manifestations, such as cognitive and personality changes, incontinence, psychosis, and parkinsonism, are the most common neurologic sequelae, and develop between 2 days and 8 months later in 10% to 30% of survivors. Many studies have provided magnitude estimates of the lesions affecting cortex, basal ganglia, globus pallidus and white matter changes within the corpus callosum and periventricular region (Chang et al., 2010; Lakhani and Bleach, 2010; Ruth-Sahd et al., 2011). Nevertheless, few investigations evaluate the relationship between the cognitive impairment and hippocampus damage after exposure to CO (Chen et al., 2013). *N*-butylphthalide (NBP), originally extracted from the seeds of Apium graveolens Linn, has displayed a broad spectrum of neuroprotective properties. It has been demonstrated that NBP could efficiently improve cognitive deficits induced by chronic intermittent hypoxia (IH)-hypercapnia exposure (Min et al., 2014), protect cells against ischemic damage via multiple mechanisms including mitochondria associated caspase-dependent and -independent apoptotic pathways both *in vitro* and *in vivo* (Li et al., 2010; Wang et al., 2014), maintain mitochondrial function and balance the expressions of anti-apoptosis genes and pro-apoptosis genes. Meanwhile, the experimental investigations have revealed that NBP administration at the dosage of (15–160 mg/ kg) was safe and reliable via oral or intraperitoneal injection (Xiong et al., 2012; Diao et al., 2014). Moreover, NBP, to some extent, could also participate in the activation of Keap1-Nrf-2/antioxidant response element (ARE) signaling pathway, and thus play neuroprotective roles against brain damage after acute CO poisoning (Li Q. et al., 2015). In the present study, we aimed to investigate the underlying mechanisms of cognitive dysfunction in rat models after exposure to CO, and evaluate the feasibility of NBP treatment on the structural and functional impairment of hippocampus induced by acute severe CO poisoning.

## Materials and Methods

### Ethics Statement

Total of 120 adult healthy male Sprague-Dawley rats (7∼8 weeks, weighing (230 ± 20) g were supplied by Qingdao Academy of Medical Sciences, China. All animal experiments were carried out in strict accordance with the regulations for the Care and Use of Laboratory Animals of the National Institute of Animal Health and the Guidance by the ethics committee of Qingdao University (animal welfare assurance number: 14-0027, Bi et al., 2016), and all possible efforts were made to minimize the pain and discomfort of each animal in accordance with the Animal Care and Use Program Guidelines of China.

### Subjects and Groups

In the present study, 120 rats were randomly assigned to three groups: a normal control group (NC group, *n* = 40), a CO poisoning group (CO group, *n* = 40) and an NBP treatment group (CO+NBP group, *n* = 40). Prior to experiments, all rats were housed in a temperature-controlled environment with a 12-h light/dark cycle for 7 days, and had free access to food and water throughout the experiment. Rats in CO group and CO+NBP group suffered from CO exposure to establish an animal model in the animal chamber as described previously (Li et al., 2016), while those in normal control group were permitted to breathe fresh air simultaneously. The subjects with coma, high HbCO concentration (≥40%) during CO inhaling and then conscious restoration after a breath of fresh air were considered as the successful models of acute severe CO poisoning. As a result, the conscious recovery time was (28.6 ± 8.8) min in CO group, and (28.5 ± 8.6) min in CO+NBP group, and there was no significant difference between the two groups (*P* > 0.05). During the whole experiment, rat core temperature was maintained at 36 ∼ 37°C using a heated blanket. Three cases were excluded in the final experimental statistics because of continued coma (one rat) or low HbCO concentration (two rats); meanwhile, another three rats of successful models were admitted in the experiment to perform the following tests.

### Treatment and Intervention

*N*-butylphthalide (chemical formula: C_12_H_14_O_2_, molecular weight: 190.24, purity: 100%) was granted by Shijiazhuang Pharmaceutical Co., Ltd., China. All rats received hyperbaric oxygen therapy within 10 min after conscious restoration (Liu W.C. et al., 2016). Rats in CO+NBP group were administrated 6 mg/100 g NBP by gavage using a stomach tube at 2 h after CO exposure additionally, twice a day for 1 day to 1 month till sacrificed (Li J. et al., 2015; Li Q. et al., 2015), and those in CO group and NC group were given the same dosage of pure olive oil as placebo at the same time.

### Evaluation of Neurological Behavior

#### Morris Water Maze Task

Morris water maze task (Shanghai soft Information Technology Co., Ltd., Model number: XR-XM101) was designed to study spatial learning and memory in all rats enrolled in the present experiment using the method described previously (Ueno et al., 2009). A platform was submerged into a tub (diameter = 130 cm; height = 50 cm; depth = 30 cm) of opaque water. The walls in the room around the water maze were covered with black cloth to create a covered area of 4-by-6 m. Two distal cues were fixed on the black walls. The animals were placed into the water from different locations at the beginning of each trial and performed four trials per session twice a day for 4 days before neurological behavior test. EthoVision XT 9 Software Analysis System was used to record the swimming route and the escape latency to finding the platform in detail. Three rats were removed from the experimental statistics because of their average escape latency far away from others, and another three rats met the experimental requirements were supplemented to the appropriate group. The average escape latency and the number of crossing platform were calculated on days 1, 3, 7, at 2 weeks and 1 month after exposure to CO. Data were expressed as the average values of four trials for each rat in different groups.

#### Shuttle Box Experimental Score

The change of learning and memory ability in the experimental rats was recorded by the active avoidance response (AAR) index established in the condition. Infrared ray will be emitted from the left and right sides of a shuttle box (model number:10080116012), respectively. If the animal locates in the center of the box at the beginning of the experiment, the shuttle test will end when it shades any light beam from either the left or right sides. If the animal stands in the left box, the test will not terminate until it shades the light beam from the right, and vice versa. Animal finishes the shuttle during the buzzer known as the AAR, while it is called passive avoidance response (PAR) in the electrical stimulation stage. Rats were first handled for 5 min per day to acclimate in the behavioral test room for 1 week prior to the start of behavior testing. In this study, the capacity of learning and memory was expressed as the ratio of AAR, that is, the rate of the completed times of ARR to the total times of test (50 times).

### Pathological Changes in Hippocampus

#### Preparation of Paraffin Sections and Hematoxylin-Eosin (HE) Staining

Four rats in each subsection were deeply anesthetized by intraperitoneal injection of 3% pentobarbital, and were perfused with 0.9% sodium chloride and 4% formaldehyde solution 200 ml transcardially at different time points mentioned above. Immediately upon harvest, brain tissues were taken out from skull, post-fixed in 4% formaldehyde for 2 h, immersed in double distilled water for 4 h, and dehydrated in gradient ethanol, transparented in dimethyl benzene, finally embedded in paraffin. Coronal sections were cut at 7 μm thicknesses through the hippocampus consecutively with a microtome (LEICA-RM 2015, Shanghai Leica Instruments Corporation, China) and adhered on the slides prepared with poly-L-lysine, then stored at 4°C. Paraffin sections were stained with HE solution as general procedure and pathological changes of the different areas (including CA1 and CA3) in hippocampus were observed under a light microscope.

#### Transmission Electron Microscopy (TEM)

To observe the ultrastructural changes of hippocampus in rats after exposure to CO, TEM was applied in the present study. Four animals in each group were deeply anesthetized and the hippocampus tissues were separated from the whole brain carefully and rapidly in a matrix surrounded by cold ice and cut into 1 mm × 1 mm × 1 mm pieces, then immersed in a fixative solution (2.5% glutaraldehyde in 0.1 mmol/l sodium cacodylate, pH 7.4) for 3 h at room temperature. After post-fixation in 1% Osmium tetroxide (pH 7.4) for 2 h at 4°C, the hippocampal pieces were embedded in epoxy resin Epon 812 and cut into ultrathin sections of 50 nm using an ultramicrotome (Leica EM UC6, Germany) on polyvinyl formal at 4°C for preservation. The slices were then immersed in the saturated alcohol solution containing 3% acetic acid uranium (pH = 3.5) in a clean culture dish and dyed for 30 min, followed by 6% lead citrate solution for the ultrastructure observation under a TEM (JEM-1200EX, Japan).

#### Golgi Staining

Four rats in each group were deeply anesthetized and perfused transcardially with sodium chloride and formaldehyde solution at the indicated time as described above, and then the whole brain were taken out, immersed thoroughly in Golgi mordant dyeing for 7 days at room temperature, followed by immersion in 30% sucrose solution for 48 h at 4°C in a dark environment. The brain tissue was cut into slices at 100 μm thickness and mounted on the anti-off load glass section for Golgi staining. Sections were rinsed thoroughly with triple-distilled water and aqueous ammonia solution (ammonia: distilled water 3:1) for 10 min and then in 1% sodium thiosulfate solution prepared freshly for another 10 min away from light at 26°C. Under a 1000-fold-light microscope, the number of dendritic spines of neurons in CA1 was calculated and analyzed in three brain slices of each rat. The amount of dendritic spines per 10 μm acted on behalf of the dendritic spine density.

### Immunofluorescence Staining

The paraffin sections were used to observe the expressions of calpain 1 and CaMK II positive cells by immunofluorescence staining assay, too. The monoclonal antibodies of the two target proteins were granted by Santa Cruz Company. The sections were blocked with sealing buffer (5% normal goat serum and 0.1% Triton X-100 in PBS) for 1 h and incubated with primary antibodies for 2 h at 37°C, (anti-calpain 1, dilution 1: 400; anti- CaMK II, dilution 1: 150), followed by fluorescence secondary antibody. All slides were observed under a fluorescence microscope (Leica, Heidelberger, Germany) in four non-overlapping fields randomly in hippocampus. The optical density (OD) value of positive cells was analyzed using Leica Qwin image processing and analysis system.

### Western Blot Analysis

Four rats in each group were deeply anesthetized as described above and then the hippocampal samples were separated by SDS-polyacrylamide gel electrophoresis (PAGE) and subsequently transferred to polyvinylidene fluoride (PVDF) membranes (Millipore, Billerica, MA, USA). After blocking in Tris-buffered saline and Tween 20 solution (TBST) containing 10% skimmed milk powder for 1 h, the PVDF membranes were incubated with primary antibodies (calpain 1 dilution 1: 550; CaMK II dilution 1: 500)for 50 min and horseradish peroxidase (HRP)-conjugated secondary antibody overnight at 4°C. Finally, the membranes were washed fully with PBS and developed in X optical film according to the manufacturer’s instructions. The absorbance (A) value of target proteins was analyzed by Bio-Rad 2000 gel imaging system and Quantity one software. The expression level of β-actin in the same sample, as an internal reference, was also detected to normalize the relative A values of target proteins.

### Statistical Analysis

Data were presented as mean ± SEM and analyzed using the Graph Prism Program, Version 5.0 (GraphPad Software, Inc., La Jolla, CA, USA). Differences in the parameters were evaluated using one-way analysis of variance (ANOVA) and least significant difference (LSD) *t*-test. Values less than 0.05 were considered statistically significant.

## Results

### Neurobehavioral Changes of Rats in Each Group

In the experiments of positioning navigation and space exploration, we observed that the average escape latency was significantly prolonged in both CO group and CO+NBP group in comparison to that of NC group (*P* < 0.05, **Table 1**). Meanwhile, we also noted that the number of crossing platform in both CO group and CO+NBP group was obviously decreased, and there was a statistical difference compared with that in NC group (*P* < 0.05). The average escape latency in CO+NBP group was shorter than that of CO group, and the number of crossing platform was slightly increased. The difference between the two groups was extremely significant at a late stage of CO poisoning (>1 weeks, *P* < 0.05), yet no significant difference was found at an early stage of poisoning (<3 days, *P* > 0.05). These results suggested that acute CO poisoning could decrease the ability of spatial learning and memory in rats, which may be closely related to the duration of CO exposure. CO+NBP could obviously improve the learning and memory function of rats, and the neuroprotective effect might last for at least 1 month after CO poisoning.

**Table 1:** Differences in the average escape latency and the number of crossing platform in Morris water maze performance in different groups.

		*n*	1 day	3 days	7 days	2 weeks	1 month
NC group	Escape latency (s)	4	44.51 ± 3.51	42.33 ± 3.26	38.42 ± 3.09	36.10 ± 2.67	26.23 ± 2.14
	Number of crossing	4	6.58 ± 0.47	6.68 ± 0.47	7.16 ± 0.51	7.32 ± 0.50	7.42 ± 0.52
CO group	Escape latency (s)	4	55.63 ± 4.28^∗^	53.16 ± 4.25^∗^	49.11 ± 3.90^∗^	45.53 ± 3.52^∗^	39.47 ± 3.11^∗^
	Number of crossing	4	1.32 ± 0.11^∗^	1.32 ± 0.08^∗^	1.32 ± 0.09^∗^	0.98 ± 0.10^∗^	0.98 ± 0.09^∗^
CO+NBP group	Escape latency (s)	4	55.32 ± 4.13^∗^	52.6 ± 4.07^∗^	40.3 ± 3.37^∗#^	38.8 ± 3.12^∗#^	30.1 ± 2.96^∗#^
	Number of crossing	4	1.34 ± 0.13^∗^	1.33 ± 0.10^∗^	1.56 ± 0.11^∗^	2.83 ± 0.18^∗#^	3.21 ± 0.21^∗#^

*^∗^Compared with NC group, *P* < 0.05; ^#^compared with CO group at the same time point, *P* < 0.05, *F* = 10.317 ∼ 17.836.*

**FIGURE 1:**
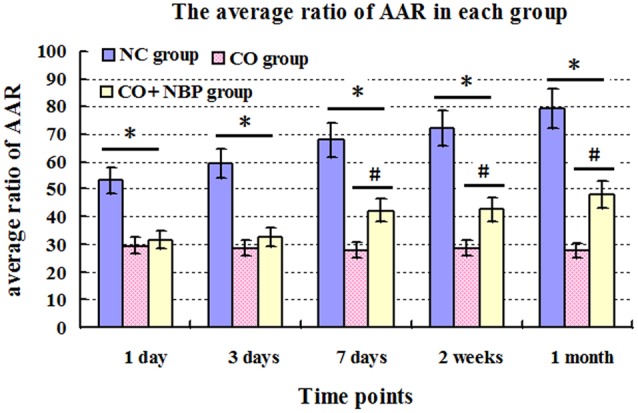
**The average ratio of active avoidance response (AAR) in NC, CO poisoning and CO+NBP treatment groups.** The average ratio of AAR in rats in CO group was notably decreased compared with those in NC group (*n* = 4, ^∗^*P* < 0.05). After administration of NBP, the average ratio of AAR was significantly increased, especially at a late stage of CO poisoning (>7 days), and accompanied by a statistical significance from 7 days to 1 month after CO poisoning compared to that of CO group (*n* = 4, ^#^*P* < 0.05). *F* = 11.528∼20.176.

The AAR of rats in CO group were notably decreased compared with that of NC group, and there were significant differences from 1 day to 1 month after CO poisoning (*P* < 0.05). However, the AAR were significantly increased after the administration of NBP, especially at a late stage of CO poisoning (>7 days), and there was statistical significance from 7 days to 1 month after CO poisoning compared to that of CO group (*P* < 0.05, **Figure 1**).

### Pathological Changes in Hippocampus in Rats

**FIGURE 2:**
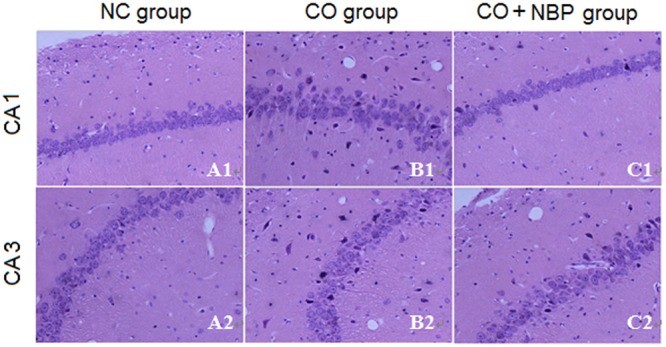
**Pathological changes of hippocampus in NC, CO poisoning and CO+NBP treatment groups using HE staining.** Neurons in CA1 region were smaller accompanied by either round or oval shape, and were aligned neatly and tightly **(A1)**, while those in CA3 **(A2)** were larger than in CA1 region and did not arrange in order. Meanwhile, few nuclear karyopyknosis and fragmentation were detected in NC samples. Neurons in both CA1 **(B1)** and CA3 **(B2)** regions in CO group were irregular shape, and part of which was pyknosis and shrinking with spindle shape. Neuronal body was roughly normal in both CA1 **(C1)** and CA3 **(C2)** regions in CO+NBP group.

Using HE staining, we found that neurons in CA1 region of hippocampal tissue in NC rats were small, with round or oval shape, and aligned neatly and tightly, while those in CA3 were larger than that in CA1, with clear outline and lightly stained nucleus, and not arranged in order. However, neurons in both CA1 and CA3 regions in CO group were irregular shape, part of which accompanied by pyknosis, even evident shrinkage with spindle-shaped morphology (**Figure 2**), suggesting that CO poisoning can obviously damage the structure of hippocampus. NBP treatment could significantly alleviate the damage of hippocampus in rats after intoxication, and neuronal bodies returned roughly normal dimension and few nuclear karyopyknosis and fragmentation were detected in NBP-treated rats.

**FIGURE 3:**
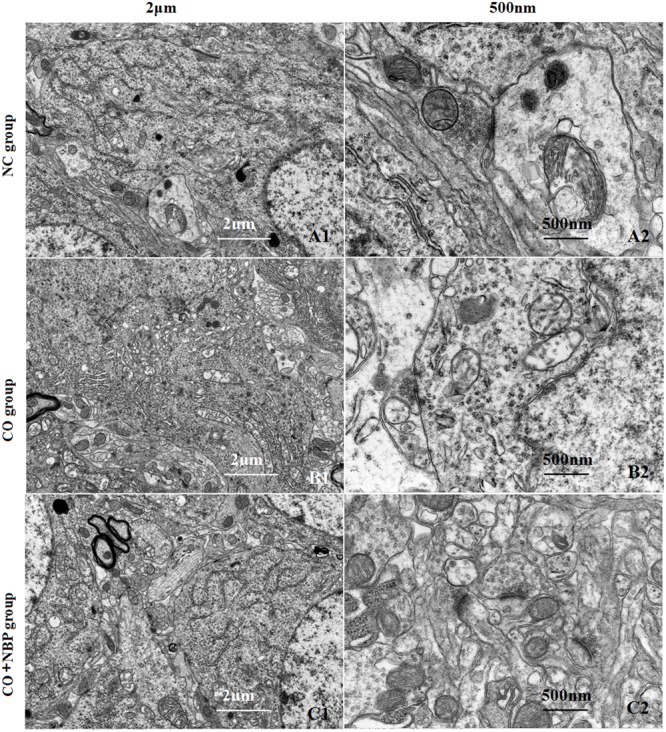
**Transmission electron microscopy (TEM) photographs of pathological changes in hippocampus in different groups.** The exterior contour of neurons in NC group was clear, with big and round nucleus and uniform chromatin on days 3 **(A1)**. The double nucleus membrane was clear and complete. Organelles were rich and scattered in cytoplasm with structural integrity **(A2)**. Hippocampal neurons were swelled, chromatin in the nucleus was condensed and marginalized, and cristae and membrane were broken. Meanwhile, mitochondria appeared to be vacuolization, and partial cell organelle dissoluted and disappeared in CO poisoning group on days 3 **(B1,B2)**. By contrast, the double-deckered nuclear membrane of neuron was clear, and mitochondria and other organelles were normal or only slightly damaged in CO+NBP group on days 3 **(C1,C2)**.

Using transmission electron microscopy (TEM), we observed that the exterior contour of hippocampal neurons in NC rats was clear, with big and round nucleus and uniform chromatin. The double nucleus membrane was clear and complete. Mitochondria, rough endoplasmic reticulum, ribosomes, Golgi bodies, and other organelles were rich, and scattered in cytoplasm with structural integrity. In contrast, hippocampal neurons were swelled, nucleus chromatin were condensed and marginalized, mitochondria appeared vacuolization, cristae and membrane were broken, rough endoplasmic reticulum dilated and the ribosomes denuded, and partial cell organelle dissolved or disappeared after CO poisoning (**Figure 3**). The damage degree of hippocampal ultrastructure in CO+NBP group was rather slighter than that of CO group. Double-deckered nuclear membrane was clear, synaptic structure was relatively complete and mitochondria were normal or only slightly swollen with few vacuoles, suggesting that NBP treatment can efficiently improve the ultrastructural damage of hippocampus induced by CO poisoning.

**FIGURE 4:**
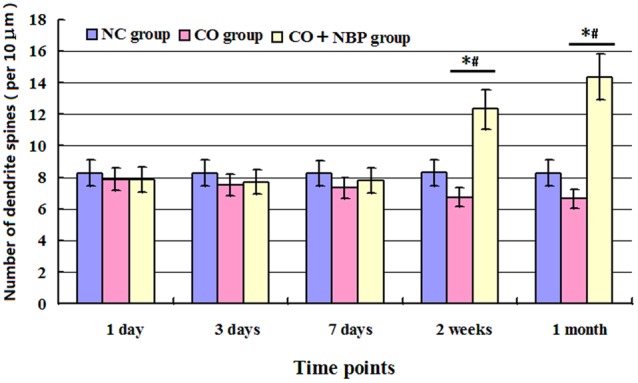
**Histogram of the number of dendrite spines per 10 μm in each group using Golgi staining.** The number of dendritic spines in hippocampal neurons in CO group was lower than that of NC group, but no statistical difference was detected at an early stage of CO poisoning compared with that of NC group (<1 week, *P* > 0.05), whereas a significant difference existed at a late stage of CO poisoning (>2 weeks, ^∗^*P* < 0.05). The administration of NBP could increase the number of dendritic spines, and it existed statistical difference as compared with CO group at a late stage of CO exposure (>2 weeks, ^#^*P* < 0.05). *F* = 13.327∼19.460.

Our study showed that the number of dendritic spines in hippocampal neurons in CO group was lower than that of NC group, but no statistical difference was detected at an early stage of CO poisoning compared with that of NC group (<1 week, *P* > 0.05), whereas a significant difference existed at a late stage of CO poisoning (>2 weeks, *P* < 0.05). Moreover, the number of dendritic spines in CO+NBP group was higher than that in CO group, and it existed statistical difference at a late stage of CO exposure (>2 weeks, *P* < 0.05, **Figure 4**).

### The Expressions of Calpain 1 and CaMK II Proteins in Rats after CO Poisoning

**FIGURE 5:**
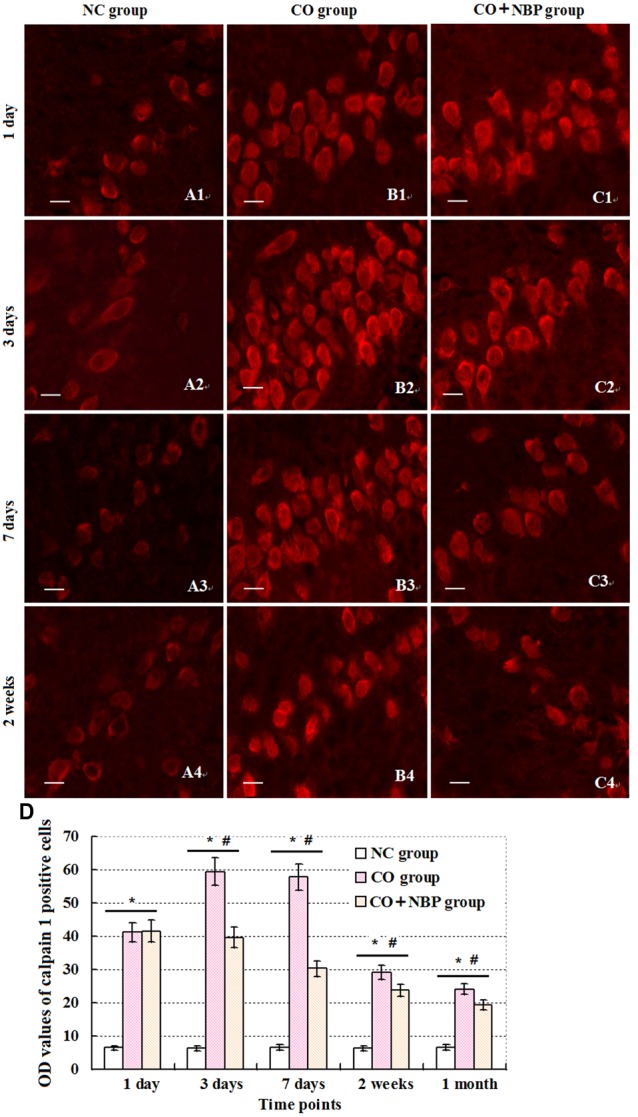
**Changes of calpain 1 in hippocampus in each group.** Calpain 1 positive cells were observed in hippocampal tissue in NC group, and mainly located in cytoplasm **(A1–C4)**. After exposure to CO, the amount of calpain 1 positive cells was gradually increased, peaked between days 3 and 7, and maintained at relatively higher levels till 2 weeks in contrast to those in NC group (*n* = 4, *P* < 0.05). In contrast, administration of NBP could significantly decrease the expression levels of calpain 1 protein in comparison to that of CO poisoning group at the same time (*n* = 4, *P* < 0.05). **(D)** Histogram of the OD values of calpain 1 positive cells in each group at different time points.^∗^Compared with NC group (*n* = 4, *P* < 0.05); ^#^compared with CO group (*n* = 4, *P* < 0.05). *F* = 11.525∼21.618. Scale bar is 30 μm.

**FIGURE 6:**
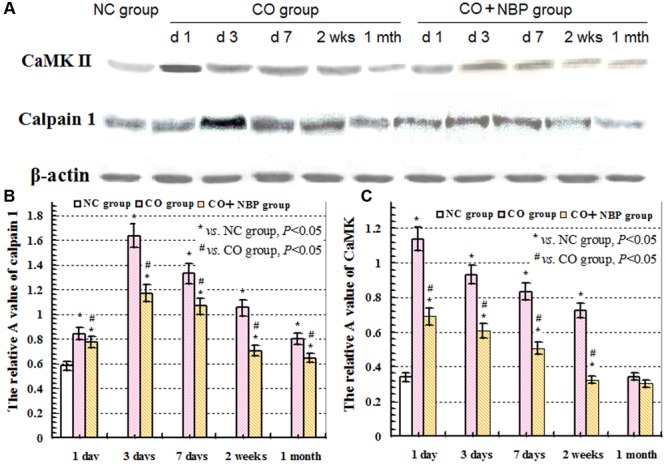
**Expressions of CaMK II, calpain 1 and β-actin proteins using western blot assay. (A)** The expression changes of CaMK II, calpain 1 and β-actin proteins in each group; **(B)** the relative A value of calpain 1 protein in different group at given time points; **(C)**: the relative A value of CaMK II protein in each group in different time points. ^∗^ *VS*. NS group, *P* < 0.05; ^#^ *VS*. CO group, *P* < 0.05, *F* = 12.436∼30.150.

Under a fluorescent microscope, a small number of calpain 1 positive cells with different sizes and red fluorescent light were observed in hippocampus in NC subjects. The positive cells were mainly located in cytoplasm, and the amount of calpain 1 positive cells was gradually increased and maintained at relatively higher levels in CO poisoning rats between 3 days and 2 weeks compared to those in NC group (*P* < 0.05). NBP treatment could notably decrease the expressions of calpain 1-positive cells, and the OD value was accordingly dropped compared with that in CO group between 3 days and 2 weeks (*P* < 0.05, **Figure 5**). Furthermore, the same results were also confirmed using western blot assay (**Figure 6**).

**FIGURE 7:**
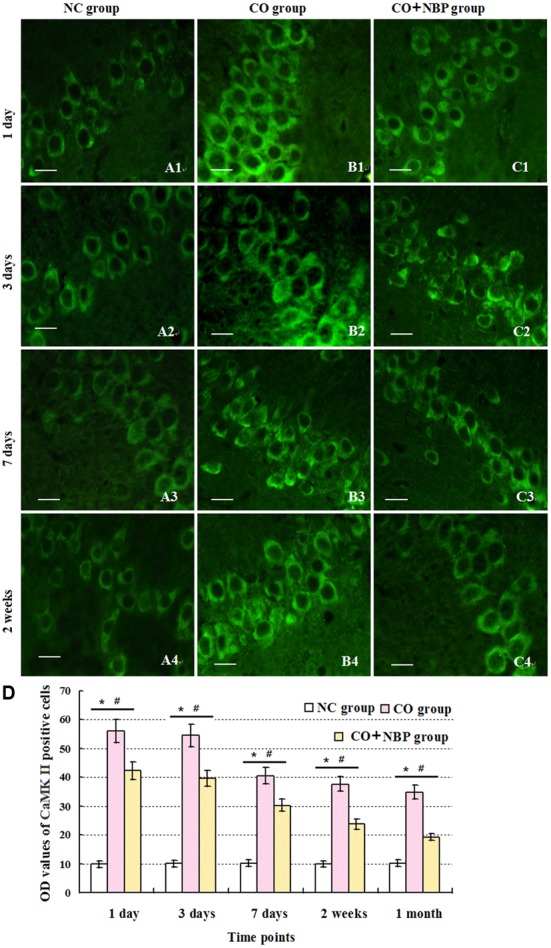
**Alterations of CamK II positive cells in each group.** CaMK II positive cells were observed in rats in NC group **(A1–A4)**, while the amount of CaMK II positive cells in CO poisoning individuals was sharply increased to the peak between days 1 and 3 with strong fluorescent values, and then decreased **(B1–B4)**. Nevertheless, it was still higher than that of NC group till 1 month (*n* = 4, *P* < 0.05). NBP administration could down-regulate the expression level of CamK II compared with that in CO group at the same times (**C1–C4**; *n* = 4, *P* < 0.05). **(D)** Histogram of the OD values of CaMK II positive cells in each group at different time points. ^∗^Compared with NC group (*n* = 4, *P* < 0.05); ^#^compared with CO group (*n* = 4, *P* < 0.05). *F* = 10.941∼22.437. Scale bar is 30 μm.

Under normal physiological conditions, a basal expression of CaMK II with weak green light in positive cells in hippocampus was observed in NC rats. After exposure to CO, the level of CaMK II increased sharply in a short time, peaked between 1 and 3 days, and then decreased. Nevertheless, it was still higher than that of NC subjects even up to 1 month. Using western blot assay, we found a similar tendency in the change of CaMK II level analyzed by immunofluorescence staining. These results revealed that the overexpression of CaMK II protein might be closely related to hippocampus damage induced by CO poisoning. In addition, we also noted that the number of CaMK II positive cells in CO+NBP treatment group was obviously lower than that in CO group at the same time (**Figure 7**, *P* < 0.05).

#### The Relationship between Calpain 1 and CaMK II

**FIGURE 8:**
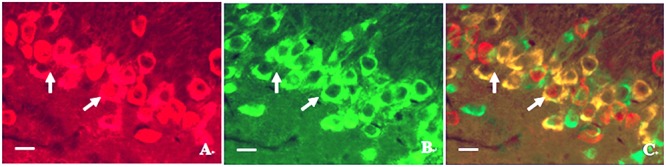
**Photographs of relationship between the locations of calpain 1 and CaMK II proteins in hippocampus under a fluorescent microscope. (A)** Calpain 1 positive cells; **(B)** CaMK II positive cells; **(C)** co-expressions of the two proteins under the same view (merged). Scale bar is 30 μm.

In order to investigate the relationship between calpain 1 and CaMK II, double immunofluorescence labeling was used in the present study. We found that although the two proteins were mainly in the cell bodies in hippocampus, some calpain 1-positive cells did not show CaMK II immunoreaction. Similarly, not all CaMK II-positive cells exerted calpain 1 immunogenicity in the same view (**Figure 8**). These results suggest that the two proteins can not only co-exist in the same cells, but also express alone in different cells. Further, we found that the expression of calpain 1 was significantly increased after CO poisoning and peaked on 3 days; while the amount of CaMK II positive cells peaked on 1 day, showing a steep rise in a short time period after exposure to CO. Subsequently, the expression levels of these two proteins were gradually decreased. The similar evidence was also assessed and validated by western blotting test, and the result of linear regression analysis clarified the positive quantitative relationship between calpain 1 and CaMK II proteins (*R*^2^ = 0.8521), indicating the two proteins would be activated successively after CO poisoning, and then influence the cognitive function of rats.

## Discussion

Carbon monoxide poisoning is the leading cause of death by poisoning in industrialized countries, and often leads to diffuse hypoxic-ischemic encephalopathy and focal cortical injury, especially in severe acute cases. Cognitive dysfunction is the most common neurological symptoms. In recent decades, many scholars focused on the mechanism, including hypoxia, lipid peroxidation, apoptosis, binding to intracellular proteins and disrupting cellular metabolism, excitotoxicity and cerebral edema, but it is still poorly elucidated about acute brain injury following CO poisoning (Han et al., 2007).

The learning and memory behavior of animal is divided into two aspects. One is based on the memory formation of fear, named passive avoidance and active avoidance, which belongs to simple memory and depends on non-condition reflex and condition reflex. The other is based on the memory formation of visual, known as spatial reference memory, which belongs to advanced memory. Shuttle box test is an important approach for quantitative determination of animal behavior changes in many neurological researches (Lalanza et al., 2015; Río-Ȧlamos et al., 2015), and belongs to the classical conditioned reflex associated with learning, while Morris water maze test mainly for the advanced intelligent activities. Together with shuttle box test and water maze test, we found that after CO poisoning, the active escape latency and the route of escape latency of rats were significantly prolonged, and the number of crossing platform was obviously decreased in rats, suggesting that CO poisoning can damage not only the advanced intelligence activities, but also the classical conditioning reflex.

Hippocampus is the main carrier of learning and memory. Animal experiments have shown that the damage of hippocampus can directly lead to learning and memory disorders. The mechanism of learning and memory is related to the electrophysiological activities of both the long-term potentiation (LTP) and the long-term depression, and the perforant pathway and other loops of hippocampus may be the anatomical basis of LTP in hippocampus (Woodard et al., 2012; Schinazi et al., 2013). Hippocampus, a component of limbic system, is also involved in the pain and emotional responses and other activities (Kim et al., 2012; Martuscello et al., 2012). There are extensive fiber connections between external and internal fibers, which are the structural basis for the complex functional implementation of hippocampus, and the structural abnormalities and pathological changes can lead to some neurological and psychiatric diseases. Golgi staining has been recognized as the most traditional and efficient method of neurological research. Using heavy metal salt staining, neurons and their small dendritic spines were significantly detected, and it is very easy to distinguish the development and death of neurons, the delivery of neurotransmitters and other aspects. In recent years, Golgi staining was used to observe the total length of dendrites and the density of dendritic spines in hippocampal neurons (Martínez-Cerdeño and Noctor, 2014; Peterson et al., 2015). The dendritic spine is closely related to neural plasticity, and the number of dendritic spines directly influences the delivery of neurotransmitters and the ability of learning and memory. In the present study, the results showed that the neuronal ultrastructure was obviously damaged, and the number of dendritic spines in hippocampal neurons was decreased in CO poisoning rats. NBP treatment could efficiently protect the structure integrity of hippocampus, benefit the development of dendritic spines, and elevate the cognitive ability of rats against CO poisoning, and the effect is more evident at a late stage of CO poisoning (>2 weeks). These results suggest that the long-term administration of NBP may be more beneficial to the recovery of learning and memory function in rats after CO poisoning.

The physiological function of hippocampus depends not only on the integrity of structure, but also on the normal quality and quantity of a variety of neurotransmitters. The calcium channels in hippocampal neurons participate in many important physiological functions of nervous system, including LTP and inhibition, learning and memory, etc. The elevated level of calcium ions in nucleus will activate the memory-related genes associated with long-term changes in postsynaptic structure, and which is the mechanism of learning and memory formation (Gurkoff et al., 2013; Nimmrich and Eckert, 2013).

**FIGURE 9:**
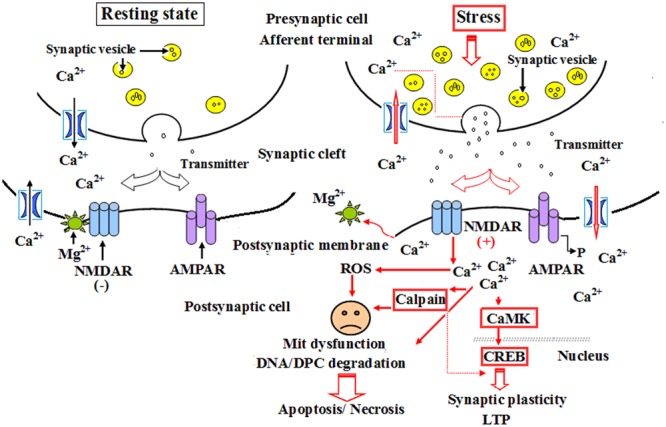
**A schematic diagram of the role of calpain 1 and CaMK II in apoptosis and long-term potentiation (LTP) after CO poisoning.** Under resting state conditions, NMDARs were interacted with magnesium ion and were not activated, the level of intracellular calcium ions was relatively low, and there was only a small amount of calpain 1 and CaMK II proteins in cytoplasm to maintain the structural and functional integrity of cells. Under the pathological circumstances, such as a strong or persistent electrical stimulation, ischemia and hypoxia, acute severe CO poisoning, NMDARs were completely activated, and the overexpressions of calpain 1 and CaMK II proteins in cytoplasm induced by calcium overload were rush into nucleus, thus participated in the process of LTP and apoptosis or necrosis.

Calpain 1 is a kind of calcium-activated intracellular protease, and ubiquitously expressed calcium-activated intracellular cysteine protease that exists in both cytosol and mitochondria. As a channel protein, calpain 1 expression is closely related to the concentration of calcium ions in neurocytes, cardiomyocytes and other cell types. Calpain-1 activation, resulting from NMDA receptor on postsynaptic membrane, mainly triggered an early neuroprotective signaling cascade potentially in a small subset of neurons in hippocampus, and was restricted to a small population of interneurons following systemic kainic acid injection (Seinfeld et al., 2016). Calpain-1 has also been demonstrated a critical role in synaptic plasticity and learning and memory, as its deletion in mice results in impairment in theta-burst stimulation (TBS)-induced LTP and various forms of learning and memory (Liu Y. et al., 2016). The continuous increase in calpain 1 expression will inevitably lead to excessive calcium influx into cells, resulting in a significant decline of cell survival rate; whereas selective calpain inhibitors have been proved the potency, efficacy and safety as possible therapeutics against abnormal synaptic plasticity and memory produced by the excess of amyloid-β, a distinctive marker of Alzheimer’s disease (Fà et al., 2015). Thompson found that the activation of mit-calpain 1 increased cardiac injury during ischemia-reperfusion (IR) by releasing apoptosis-inducing factor, sensitizing mitochondrial permeability transition pore (MPTP) opening, and impairing mitochondrial metabolism through damaging complex I. MDL-28170, an inhibitor of calpain 1, could effectively alleviate cardiac injury during IR by inhibiting both cytosolic and mitochondrial calpain 1 (Thompson et al., 2016). The results of Wang et al. (2016) demonstrated that IH increased calpain enzyme activity and reactive oxygen species (ROS) level as well as Ca^2+^ concentration, and these effects could be eliminated by a membrane-permeable ROS scavenger. Therefore, they insisted that the activation of calpains by ROS-dependent elevation of Ca^2+^ mediate human ether-a-go-go-related gene (hERG) channel protein degradation by IH (Wang et al., 2016). Our investigation showed that the abnormal expression of calpain 1 was related to the ultrastructural damage of hippocampus to some extent during CO exposure. These results were consistent with those of Fà et al. (2015) and Wang et al. (2016), but slightly different from Seinfeld et al. (2016). We conceive the different roles of calpain 1 in the process of pathological state, i.e., the slight and transient increase of calpain 1 expression may play an endogenous protection in the super early stage of CO poisoning, whereas the activation of NMDA receptor and the overload of intracellular calcium will result in the over-expression of calpain 1 protein in a bite late period after exposure to CO, and then lead to cell apoptosis/necrosis through mitochondrial-mediated signaling pathway (**Figure 9**). NBP treatment could notably decrease the expressions of calpain 1-positive cells, suggesting that NBP may efficiently protect hippocampus neurons against CO toxicity via down-regulating the expression of calpain 1 protein in brain tissue in rats followed by CO poisoning.

Calcium/calmodulin-dependent protein kinase II (CaMK II) is a major multiple functional calcium-regulated enzyme and abundant in brain tissue, especially in hippocampus, which regulates neuronal receptor- gated ion channels, calcium-dependent ion currents and the synthesis and release of neurotransmitters. It has been demonstrated that persistent activation of CaMK II is dependent on the autophosphorylation of Ca^2+^/calmodulin, and the latter in hippocampus plays a critical role in synapse formation, receptor and ion channel function, gene expression, and memory processing and neuroplasticity. Many experimental animal models revealed that formation of learning and memory, such as hippocampal-dependent spatial learning, is strongly responsible for the activity of CaMK II (Malik and Hodge, 2014). Thus, prevention of CaMK II autophosphorylation could obviously impair spatial learning and memory tasks in mutant mice (Giese et al., 1998), whereas the administration of morphine sensitization apparently increased both Ca^2+^/calmodulin- independent and -dependent activities of CaMK II in hippocampus in rat models (Kadivar et al., 2014). Moreover, Ashpole and Hudmon (2011) found that a short time suppression of the abnormal CaMK activation could reduce the mortality rate of hippocampal neurons, while the neuroprotective effect was lost, and cell death would inevitably occur if the sustained inhibitory of CaMK expression was more than 8 h, and vice versa. Nevertheless, acute morphine induction at a dosage of 5 mg/kg did not alter either CaMK II mRNA expression or CaMK II activity in hippocampus, and overexpression of CaMK II in transgenic mice resulted in the enhancement of spatial memory acquisition (Mayford et al., 1995). CaMK II, the downstream signal molecular of *N*-methyl-D-aspartate receptors (NMDARs) and cAMP- response element binding protein (CREB), plays a crucial role in inducing the formation of LTP, whose generation and maintenance need the synthesis of new proteins (Zhu et al., 2014). Thus, as a “molecular switch,” the relatively invariable activity of CaMK II in cytoplasm may be essential for cell survival and trigger LTP process and short-term memory formation. Our result showed that the level of CaMK II increased sharply during short time intervals, and still maintained at a higher level till 1 month after exposure to CO even up to 1 month. This result was identical to that of calpain 1 expression. Thus, we assumed that under normal circumstances, NMDARs were not activated due to the combination with magnesium ions, the concentration of intracellular calcium was relatively low, and there was only a small amount of calpain 1 and CaMK II proteins in cytoplasm to maintain the structural and functional integrity of cells. However, when suffered a strong or persistent stimulus, such as acute severe CO poisoning, magnesium ions were escaped from the complex and NMDARs were further completely activated. Therefore, the overexpressions of calpain 1 and CaMK II proteins in cytoplasm induced by calcium overload were rush into nucleus, eventually led to degradation of DNA and NPC, and even apoptosis/necrosis (Wang et al., 2013; Chimura et al., 2015; **Figure 9**), whereas early application of NBP can significantly reduce the expressions of calpain 1 and CaMK II proteins, suggesting that NBP may improve cognitive function and maintain neuronal survival and function via inhibiting these two target proteins in rats after CO poisoning.

In summary, the results demonstrated that NBP at the dosage of (6 mg/ 100g) was safe via oral and no side effect was found in any of the SD rats in the present study. NBP treatment can efficiently improve learning and memory function, maintain the structure integrity of hippocampal neurons, inhibit the expressions of memory-related proteins, thereby preventing rats from cognitive impairment after exposure to CO. The neuroprotective effect of NBP is involved in the down-regulation of both calpain 1 and CaMK II expression. Early application of NBP may be more conducive to the resumption of cognitive function in patients with acute CO poisoning.

## Conclusion

Based on these findings, we inferred that NBP treatment could improve the ultrastructure and cognitive function of hippocampus in rats with CO poisoning, which is associated with the down-regulation of both calpain 1 and CaMK II proteins.

## Author Contributions

Conceived and designed the experiments: QL and D-DG. Performed the experiments: X-NS, X-YD, Y-HZ, and BL. Analyzed the data: M-JB and YZ. Contributed reagents/materials/analysis tools: M-JB. Wrote the paper: QL and D-DG.

## Conflict of Interest Statement

The authors declare that the research was conducted in the absence of any commercial or financial relationships that could be construed as a potential conflict of interest.

The reviewer YG declared a shared affiliation, though no other collaboration, with the authors to the handling Editor, who ensured that the process nevertheless met the standards of a fair and objective review.
